# An Intelligent Cost-Reference Particle Filter with Resampling of Multi-Population Cooperation

**DOI:** 10.3390/s23146603

**Published:** 2023-07-22

**Authors:** Xinyu Zhang, Mengjiao Ren, Jiemin Duan, Yingmin Yi, Biyu Lei, Shuyue Wu

**Affiliations:** 1 Shaanxi Key Laboratory of Complex System Control and Intelligent Information Processing, Xi’an University of Technology, Xi’an 710048, China; 2220320137@stu.xaut.edu.cn (M.R.); 2210321191@stu.xaut.edu.cn (J.D.); yiym@xaut.edu.cn (Y.Y.); lby_zj1998@163.com (B.L.); wsy152292@163.com (S.W.); 2 Faculty of Automation and Information Engineering, Xi’an University of Technology, Xi’an 710048, China

**Keywords:** state estimation, unknown statistical characteristics of noise, cost-reference particle filter, multi-population cooperation, intelligent resample, Gaussian mutation

## Abstract

Although the cost-reference particle filter (CRPF) has a good advantage in solving the state estimation problem with unknown noise statistical characteristics, its estimation accuracy is still affected by the lack of particle diversity and sensitivity to the particles’ initial value. In order to solve these problems of the CRPF, this paper proposed an intelligent cost-reference particle filter algorithm based on multi-population cooperation. A multi-population cooperative resampling strategy based on ring structure was designed. The particles were divided into multiple independent populations upon initialization, and each population generated particles with a different initial distribution. The particles in each population were divided into three different particle sets with high, medium and low weights by the golden section ratio according to the weight. The particle sets with high and medium weights were retained. Then, a cooperative strategy based on Gaussian mutation was designed to resample the low-weight particle set of each population. The high-weight particles of the previous population in the ring structure were randomly selected for Gaussian mutation to replace the low-weight particles in the current population. The low-weight particles of all populations were resampled in turn. The simulation results show that the intelligent CRPF based on multi-population cooperation proposed in this paper can reduce the sensitivity of the CRPF to the particles’ initial value and improve the particle diversity in resampling. Compared with the general CRPF and intelligent CRPF with adaptive MH resampling (MH-CRPF), the RMSE and MAE of the proposed method are lower.

## 1. Introduction

State estimation has interested scholars around the world for a long time. It is an important problem in the fields of parameter detection, automatic control, fault diagnosis and navigation guidance [1,2,3]. A particle filter (PF) is a recursive Bayesian estimation method based on the Monte Carlo idea. This method approximates the posterior probability distribution of the system by sampling a large number of particles (random samples). The weighted sum of these particles is used instead of the high-dimensional integral for system state estimation. Due to its good solving ability for nonlinear non-Gaussian systems, the application scope of filtering technology in state estimation is greatly expanded [4,5,6]. However, the particle filter and its improved algorithms can only obtain good filtering results when the statistical characteristics of the process noise and measurement noise are known [7]. In practical applications, such as the growth of single-crystal silicon by the Czochralski method and the prediction of lithium–ion’s remaining lifetime, the system state measured by the sensor contains noise. Due to the interference of some variable factors or the external environment, the noise statistical characteristics are difficult to identify. The standard particle filter may reduce the accuracy of state estimation, which affects the analysis and control of the system [8]. In view of the fact that the statistical characteristics of the noise are unknown, the cost-reference particle filter (CRPF) was proposed for the first time [9]. This method does not need to know the statistical characteristics of process noise and measurement noise [10]. It calculates the weight of each particle by a self-defined cost function and risk function, and then resamples according to the weight. Finally, the system state is approximated by the set of resampled particles. Since the cost-reference particle filter does not need to know the statistical characteristics of the noise, it has been widely used since it was proposed. It has gradually become an effective method to solve the problem of filtering nonlinear systems with unknown noise statistical characteristics. However, the CRPF still uses traditional polynomial resampling. The particles are selected according to the weight: the particles with high weights are copied in high numbers and the particles with low weights are eliminated. Therefore, the particles are concentrated in the same area, and the number of the same particles gradually increases, resulting in the loss of offspring diversity. The particles cannot completely cover the posterior probability distribution, that is, there is a lack of particle diversity, so that the filtering results have a large deviation. In addition, the CRPF is sensitive to the initial value of the particle distribution. Filtering results of the CRPF will further deteriorate when the initial value of the particle is unknown. Therefore, how to develop an efficient and reliable resampling method under the framework of the CRPF, to further improve the diversity of particles when the initial distribution of particles is unknown, and then improve the accuracy of the CRPF, becomes an important issue in the research of the CRPF.

At present, many scholars have conducted a significant amount of research and proposed some improved resampling algorithms to solve the problem of lack of particle diversity. The paper [11] proposed a deterministic resampling strategy. Instead of blindly copying particles with high weights and discarding particles with low weights as in traditional resampling methods, this strategy replicates particles selectively. It divides particles based on their state values and weights. Therefore, the number of effective particles in this method was improved. The paper [12] proposed a particle filter algorithm based on error ellipse resampling. In this method, the particle set is divided hierarchically by defining an error ellipse, and then the particles are copied according to the level of division. These resampling methods suppress the problem of lack of particle diversity to a certain extent. However, they still adopt the traditional resampling framework and replace the abandoned particles by copying the existing particles, which cannot fundamentally solve the problem. In recent years, the development of swarm intelligence optimization algorithms has provided new ideas for improving the particle diversity. In the literature [13,14,15], the artificial fish swarm algorithm, the firefly algorithm and the self-controlled bat algorithm are, respectively, used in the particle filter, attempting to optimize all the particles in each resampling by the swarm intelligence optimization method. Although these methods have a certain effect on increasing the diversity of the particles, they will increase the complexity of the algorithm and seriously reduce its real-time performance. In addition, these methods run the risk of introducing the problem of falling into a local optimum that exists in most swarm intelligence optimization methods, which affects the estimation accuracy of the particle filter. In 1997, the paper [16] proposed that the particle filter and evolutionary algorithm have a similar structure, and introduced the crossover operation of the genetic algorithm into the particle filter to increase the diversity of the particles. The paper [17] proposed an intelligent particle filter algorithm based on a real-coded crossover and mutation strategy. This method uses a real-coded crossover and mutation strategy to increase the diversity of the particles. Based on this idea, the paper [18] proposed a sequential evolutionary filtering algorithm without traditional resampling, which improved the running speed of the algorithm. Subsequently, the paper [19] introduced selection, crossover and mutation operators into resampling, which enriched the resampling strategy based on genetic operation and further improved the particle diversity. Although the resampling method based on the crossover and mutation strategy can improve the diversity of the particles, it is difficult to obtain an ideal particle distribution due to the dynamic change in particle distribution. Therefore, the estimation accuracy of the particle filter needs to be further improved. The paper [20] proposed an adaptive resampling particle filter based on Student’s t distribution. The two subsets divided according to the weight are adaptively crossed. After that, some particles are mutated randomly to improve the diversity of the particles. The paper [21,22] also proposed an adaptive genetic particle filter and enhanced mutation particle filter, respectively. They improved the efficiency of particle resampling by adaptively adjusting the mutation rate. The above methods introduce the crossover and mutation operations of the genetic algorithm to increase the diversity of the particles. Although this is of great significance for the development of resampling algorithms, it seriously affects the computational complexity and realizability of the algorithm. As the resampling strategy improves, the number of parameters to set increases. The paper [23] proposed an adaptive Metropolis–Hastings (M–H) resampling algorithm, which introduced the accept–reject mechanism of M–H into resampling. It adaptively selected Gaussian mutation or crossover of the high weight and low weight to resample particles according to the particle distribution, effectively improving the diversity of the particles. The above methods have made good progress in improving resampling, but they are all based on the traditional particle filter and have not been extended to the CRPF. Therefore, they still assume that the particles’ initial value and noise distribution are known. However, in practice, the particles’ initial value and noise distribution are often not accurately obtained, which affects the estimation accuracy. Since the particle filter and swarm intelligence evolutionary algorithm have the same structure, the latest research results in swarm intelligence methods can provide a new development direction for the research of resampling strategy [24], such as the multi-swarm cooperation mechanism. The multi-population cooperative mechanism means that the population is divided into several populations in the evolution process, each population evolves independently and then the information is shared among the populations. Thus, the convergence speed of the algorithm is improved and the algorithm is kept from falling into local optimum. The multi-population cooperation mechanism provides a new idea for further improving the diversity of particles and solving the problem of low estimation accuracy, when the initial values of the particles and the statistical characteristics of the noise are unknown.

Therefore, this paper proposes an intelligent resampling method based on multi-population cooperation, which improves the previous resampling method and further improves the particle diversity. The new resampling method is applied in the cost-reference particle filter (CRPF) to solve the problem of lack of particle diversity and sensitivity to the particles’ initial value in the CRPF, which is good at estimating the system state when the statistical characteristics of noise are unknown. Firstly, the particles are divided into several independent populations, and each population performs importance sampling using the distribution of different initial values. Then, the particles of each population are sorted according to the weight from large to small, and the golden section ratio is used to divide the particle set into three parts: high, medium and low weight; while retaining the set of particles with high and medium weights, Gaussian mutation is performed on the particles with high weights with a certain probability. By using the cooperative strategy, the low weight particles in the current population are replaced by the high weight particles after mutation in the previous population, so as to realize the resampling of the low weight particle set in turn. In this paper, a new method is proposed to realize information sharing among populations through the ring coordination mechanism, which makes the particles in the population closer to the posterior probability density of the state. The superimposed Gaussian mutation effectively increases the diversity of the particles in the resampling process. The final state estimate is obtained according to all particles and their corresponding weights. Finally, the effectiveness of the proposed method was verified by common one-dimensional and multidimensional models.

## 2. Materials and Methods

Suppose the nonlinear discrete system model is described as follows:

(1) $$x_{k}=f\left(x_{k-1}\right)+w_{k}$$

(2) $$y_{k}=h\left(x_{k}\right)+v_{k}$$

where, $x\in R^{n_{x}}$ and $y\in R^{n_{y}}$ are system state vector and observation vector at time *k*. $w\in R^{n_{x}}$ is process noise, $v\in R^{n_{y}}$ is observation noise, $f\left(x_{k-1}\right)$ and $h\left(x_{k}\right)$ are bounded nonlinear mappings, respectively.

Unlike the traditional particle filter, the CRPF does not need to know the statistical characteristics of noise when estimating the state. Instead, it proposes a cost function and a risk function, and finally calculates the weight of the particle by them. The cost function of the *i*-th particle $x_{k}^{i}$ at time *k* is defined as follows:

(3) $$C_{k}^{i}=\lambda C_{k-1}^{i}+\Delta C_{k}^{i}$$

(4) $$\Delta C_{k}^{i}=\Delta C\left(x_{k}^{i}|y_{k}\right)={∥y_{k}-h\left(x_{k}^{i}\right)∥}^{q}$$

In the formula, 0≤λ<1 is the forgetting factor, q≥1. The risk function of the *i*-th particle at time *k* is defined as follows:

(5) $${ℜ}_{k}^{i}=\lambda C_{k-1}^{i}+{∥y_{k}-h\left(f\left(x_{k-1}^{i}\right)\right)∥}^{q}$$

According to Equations (3)–(5), when the particle is closer to the true state value, the cost due to the estimation error is smaller. This indicates that the risk to be taken when selecting the particle is smaller, so the probability that the particle is selected to be retained is larger. That is, its weight is higher. The algorithm steps for the standard CRPF are as Algorithm 1.

According to the above steps of the CRPF algorithm, the process noise and measurement noise of the system are not used in the whole process of system state estimation. Instead, it is achieved through the cost function and risk function defined by the CRPF algorithm. Therefore, compared with the conventional particle filter, the CRPF has a significant advantage in solving the state estimation problem with unknown noise statistical properties.

**Figure 1:**
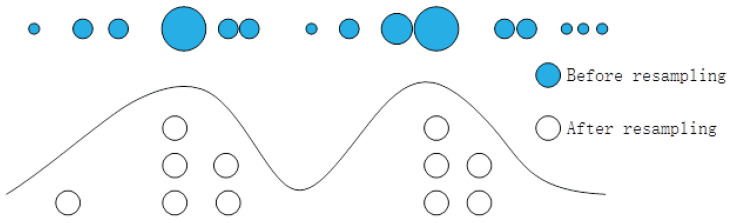
Schematic diagram of traditional resampling particle distribution.

Although the CRPF has a good filtering effect when the statistical characteristics of noise are unknown, it still uses the traditional resampling method. It copies high-weight particles and discards low-weight particles, so the problem of lack of particle diversity also exists in the CRPF. When the particles with high weight are copied and most particles with small weight are discarded, this leads to the result of state estimation deviating from the real state, due to the entire set of particles being unable to completely cover the posterior distribution, as shown in Figure 1. At the same time, the initial value of the particle cannot be accurately obtained in practical problems, which makes the problem more prominent in the estimation process of the CRPF. Therefore, how to improve the resampling algorithm of the CRPF, so that it can effectively suppress the lack of particle diversity and improve the estimation accuracy when the particles’ initial value is not accurate, has become a key issue in the study of the CRPF algorithm.
**Algorithm 1** The algorithm steps for the standard CRPF.(1)Initialize *k* = 0
(1)N particles are extracted from the initial distribution $p\left(x_{0}\right)$, the initial cost is set to 0;(2)The initial particle set is ${\left\{x_{0}^{i},C_{0}^{i}\right\}}_{i=1}^{N}$, where $p\left(x_{0}\right)$ is uniformly distributed;
(2)Resample
(1)The risk function value of the particle is calculated by Formula (5);(2)The weight of the particle is calculated from the risk function value according to the following formula:
(6)$$w_{k}^{i}=\frac{{\left({ℜ}_{k}^{i}\right)}^{-\beta }}{\sum_{i=1}^{N}{\left({ℜ}_{k}^{i}\right)}^{-\beta }}$$
where β>1;(3)Polynomial resampling is performed on the particle according to the weight value, and the particle set after resampling is ${\left\{{\bar{x}}_{k}^{i},{\bar{C}}_{k}^{i}\right\}}_{i=1}^{N}$;
(3)Update
(1)Carry out particle update according to the following formula:
(7)$x_{k}^{i}∼N\left(f\left({\bar{x}}_{k}^{i}\right),\Sigma \right)$(2)Update particle cost by Formula (3) and (4);(3)Calculate the updated particle weight according to the following formula:
(8)$$w_{k}^{i}=\frac{{\left(C_{k}^{i}\right)}^{-\beta }}{\sum_{i=1}^{M}{\left(C_{k}^{i}\right)}^{-\beta }}$$
(4)State estimationThe estimated system status is
(9)$${\hat{x}}_{k}=\sum_{i=1}^{N}x_{k}^{i}w_{k}^{i}$$

## 3. Intelligent CRPF Based on Multi-Population Cooperation

### 3.1. Multi-Population Cooperative Intelligent Resampling Mechanism Based on Ring Structure

**Figure 2:**
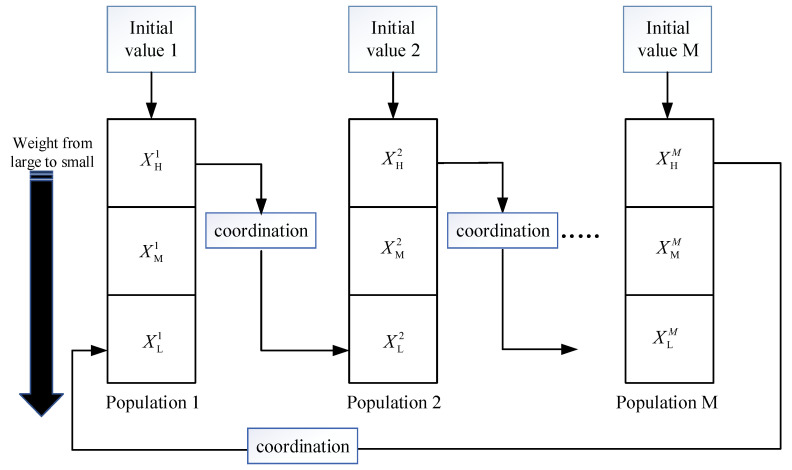
Intelligent resampling mechanism based on ring structure.

In order to solve the problem of low estimation accuracy due to the lack of particle diversity in the CRPF when the initial value is not accurate, an intelligent cooperative resampling mechanism based on ring structure is proposed in this paper. The structure of the intelligent cooperative resampling mechanism is shown in Figure 2. The particles are extracted from the importance distribution of M different initial values, and the extracted particles form a population with N particles which are mutually independent. The particles in each population were sorted according to their weights and divided into three parts according to the golden proportion. The golden proportion is strictly proportional, artistic and harmonious, and is considered to be the most ideal proportion in architecture and art. The first 0.382 N particle set is $X_{H}^{s}$ with a larger weight, the 0.382 N particle set is $X_{L}^{s}$ with the smallest weight and the remaining particle set is $X_{M}^{s}$ with a middle weight, where s=1,2,…M. The high weight particle set $X_{H}^{s}$ and medium weight particle set $X_{M}^{s}$ with a larger weight in each population are directly retained. However, since the particles in $X_{L}^{s}$ have little influence on the estimated results, all the particles in it are resampled using the cooperative strategy. In order to ensure the particle resampling order and particle diversity, the cooperation strategy between various swarms is completed based on the ring structure. When the low-weight particles of the s-th population are resampled, the high-weight particles of the s−1 population are selected for cooperation. When s = 1, then the particles are drawn from the set of high-weight particles of the M-th population for cooperation, and the details of the cooperation strategy will be described in Section 3.2. After resampling all the particles with low weights in M populations, the weights of the particles are recalculated. The weighted sum of all particles in each population is used to obtain the state estimate value, and then the final state estimate value of the whole particle set is obtained.

Different from the traditional resampling methods used in the CRPF, new resampling mechanisms are proposed in this paper. Instead of simply copying the high-weight particles to replace the low-weight ones, this method adopts an intelligent cooperative strategy to improve the diversity of the particles. Compared with the previous intelligent resampling methods, the intelligent cooperative resampling mechanism adopts a multi-population ring cooperative structure. Each population uses the importance distribution of different initial values to generate particles, which reduces the sensitivity of the particle filter to the initial values. In addition, the collaborative strategy was used to exchange high-weight particle information between different populations, which improved the information sharing ability of the particles and effectively improved the diversity of the particles. At the same time, the traditional resampling step is canceled to reduce the complexity of the algorithm.

### 3.2. Cooperative Strategy Based on Gaussian Mutation

Since the particles in the high weight and medium weight particle sets have large weights and are all effective particles, these sets must be retained when resampling the particles. Only the low weight particles in each population are resampled by the collaborative strategy. This can not only improve the efficiency of resampling, but also increase the diversity of the particles. However, the traditional resampling method can no longer be used for cooperative operation. Copying particles with high weight and directly replacing particles with low weight will cause the same particles to be copied in large numbers, resulting in the serious loss of particle diversity and the reduction in particle filtering accuracy.

In this paper, a collaborative strategy based on Gaussian mutation is designed to resample low-weight particles in each population, avoiding blindly copying high-weight particles to improve the diversity of the particles. This strategy is based on the ring structure of Section 3.1. Particles are randomly selected from the high-weight particle set of the previous population, and Gaussian mutation is performed on them. Then, the low-weight particles of the local population are replaced with the mutated particles.

Gaussian mutation is calculated as follows:

(10) $$x_{kS}^{ls}=x_{kH}^{ns-1}+q^{*}\lambda$$

In Formula (10), $x_{ks}^{ls}$ is the particle after mutation at time k, $l=1,2,\ldots ,N_{kL}^{s}$ and $N_{kL}^{s}$ are the particle numbers of the low-weight particle set in the s-th population at time k, $x_{kH}^{is-1}$ is the particle number of the high-weight particle set randomly selected from the (s−1)-th population at time k, $n\in \left\{1,2,\ldots ,N_{kH}^{s-1}\right\}$ and $N_{kH}^{s-1}$ are the particle numbers of the high-weight particle set in the (s−1)-th population at time k, while λ∼N(0,1) and *q* are the mutation rate.

According to the above equation, when *q* is set appropriately, the particle with Gaussian mutation must be located near the particle with high weight. Different from the existing high-weight particles, this method can effectively improve the diversity of the particles and ensure the quality of the whole set of particles. At the same time, *q* should not be too large; otherwise, the newly generated particles will be far away from the high probability region, and then reduce the accuracy of filtering. The organic integration of cooperative strategy and ring structure ensures that the information of high-weight particles generated by different importance distributions of each population is shared. After several iterations, the particles generated by the importance distribution far away from the true posterior distribution are gradually abandoned, and more and more particles close to the posterior distribution are retained. This strategy effectively improves the diversity of the particles, thereby reducing the impact of inaccurate initial value settings on the state estimation results.

### 3.3. Steps of Intelligent CRPF Algorithm Based on Multi-Population Cooperation

The multi-population cooperative intelligent resampling proposed above is introduced into the CRPF algorithm to obtain the intelligent CRPF based on multi-population cooperation. The algorithm steps are as Algorithm 2.
**Algorithm 2** Steps of Intelligent CRPF Algorithm Based on Multi-Population Cooperation.(1)Initialize
(1)Set the population number M, the particle number N of each population and the uniform distribution parameter $\left[a_{s},b_{s}\right]$ of each population, s=1,2,…,M;(2)Randomly extract the initial particle of each population from the uniform distribution $x_{0}^{is}$, i=1,2,…,N; and set the initial value corresponding to each particle to 0, that is $C_{0}^{is}=0$, to obtain the particle-cost set ${\left\{x_{0}^{is},C_{0}^{is}\right\}}_{i=1}^{N}$;
(2)Resample
(1)The risk function ${ℜ}_{k}^{is}$ of each particle is calculated according to Formula (5), and the particle weight $w_{k}^{is}$ is calculated according to Formula (6);(2)Arrange the particles in each population according to the weight from large to small, and retain $X_{kH}^{s}$ and $X_{kM}^{s}$ in each population;(3)Resampling the particle $x_{kL}^{is}$ in the low-weight particle set $X_{kL}^{s}$ of each population according to Formula (10) and Section 3.2 to obtain the particle set $X_{kS}^{s}$. If s=1, the high-weight particle is extracted from $X_{kH}^{M}$ for resampling according to Formula (10);(4)The high-weight particle set $X_{kH}^{s}$ and medium-weight particle set $X_{kM}^{s}$ of each population are combined with the resampling particle set $X_{kS}^{s}$ to obtain the new set of each population, while each population remains independent;
(3)Update
(1)The resampled particles are updated according to Formula (7), the updated particle cost $C_{k}^{is}$ is calculated according to Formulas (3) and (4) and the updated particle weight $\omega_{k}^{is}$ is calculated according to Formula (8);(2)Calculate the estimated value ${\hat{x}}_{k}^{s}$ of each population according to Formula (9);(3)Finally, calculate the final state estimate of the system according to the estimated value ${\hat{x}}_{k}^{s}$ of each population according to the formula below:
(11)$${\hat{x}}_{k}=\frac{1}{M}\sum_{s=1}^{M}{\hat{x}}_{k}^{s}$$


## 4. Results and Discussion

In order to verify the effectiveness of the proposed method, three simulations were designed. The standard CRPF and the adaptive MH resampling-CRPF (MH-CRPF, Intelligent CRPF) are used as comparison algorithms, and each experiment was independently repeated 100 times. The three simulation models are the mathematical model of one-dimensional non-stationary economic growth [17,18], the one-dimensional nonlinear univariate time series model [3,21] and the lithium–ion battery remaining life prediction model [1], respectively. Due to their strong nonlinear characteristics, these models are widely used to verify the effectiveness of the particle filter. Meanwhile, root mean square error (RMSE) and mean absolute error (MAE) are selected to evaluate the performance of the algorithm [25]. RMSE and MAE are, respectively, calculated according to the following formula:

(12) $$RMSE=\sqrt{\frac{1}{KL}\sum_{i=1}^{K}\sum_{k=1}^{L}{\left({\hat{x}}_{k,i}-x_{k,i}\right)}^{2}}$$

(13) $$MAE=\frac{1}{KL}\sum_{i=1}^{K}\sum_{i=1}^{L}\left|{\hat{x}}_{k,i}-x_{k,i}\right|$$

where *K* represents the number of repeated experiments, *L* is the length of time series, ${\hat{x}}_{k,i}$ and $x_{k,i}$ are, respectively, the estimated value and the real value of the system state at the time *k* in the *i*-th simulation.

### 4.1. Mathematical Model of One-Dimensional Non-Stationary Economic Growth

The one-dimensional non-stationary economic growth mathematical model has the characteristics of being strong nonlinear and bimodal, and it is difficult to estimate its system state, so the model is widely used to verify the effectiveness of the particle filter. Its model is described as follows:

(14) $$x_{k}=\frac{1}{2}x_{k-1}+\frac{25x_{k-1}}{1+x_{k-1}^{2}}+8\cos\left[1.2\left(k-1\right)\right]+w_{k}$$

(15) $$y_{k}=\frac{1}{20}x_{k}^{2}+v_{k}$$

where $w_{k}∼N\left(0,\sigma_{w}^{2}\right)$ and $v_{k}∼N\left(0,\sigma_{v}^{2}\right)$ are process noise and observation noise, respectively.

In this simulation, $\sigma_{v}^{2}=1$, $\sigma_{w}^{2}$ is the random number subject to uniform distribution U(0,9), and the initial value of $x_{k}$, $x_{0}$ is the random number subject to uniform distribution U(0,12). Each independent repeated experiment is randomly generated. The population number of this method is M = 3, and the particle number of each population is N = 80. The initial state values of the three populations are $x_{0}^{1}∼U\left(0,4\right)$, $x_{0}^{2}∼U\left(4,8\right)$ and $x_{0}^{3}∼U\left(8,12\right)$, respectively, and the variation rate of Gaussian mutation is 2. The particle numbers of the standard CRPF and MH-CRPF are both 240, and the initial particle values are random numbers that follow uniform distribution U(0,12).

**Figure 3:**
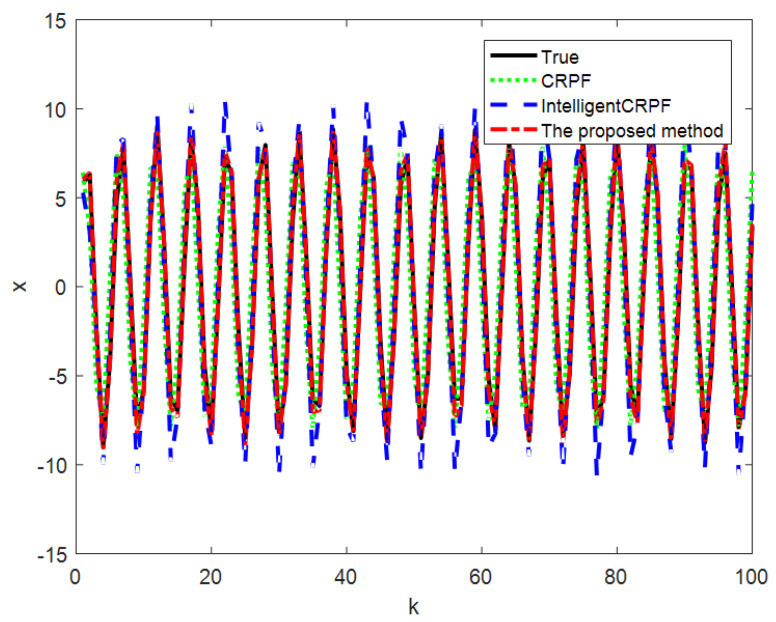
Comparison of three methods for state estimation of model Section 4.1.

**Figure 4:**
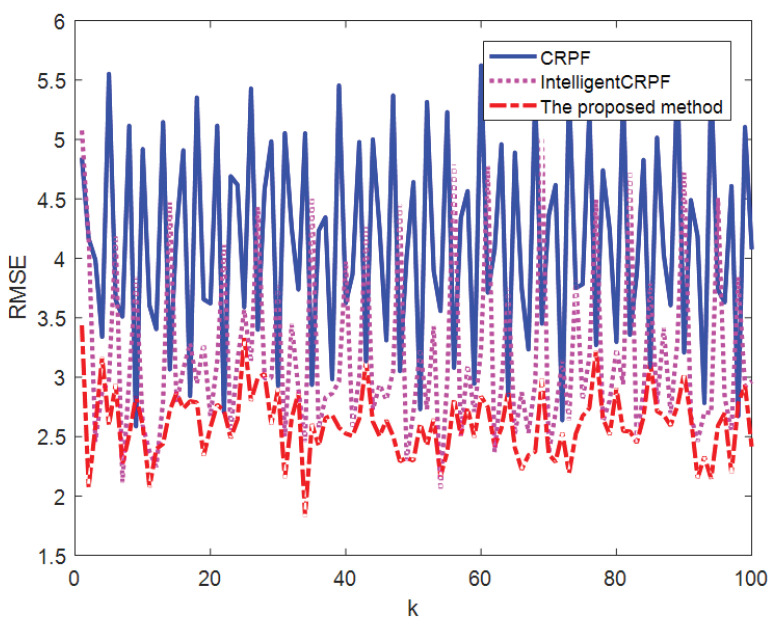
RMSE of state estimation results of three methods of model Section 4.1.

Figure 3 shows the comparison of the average value of the 100-time state estimation results of the mathematical model of one-dimensional non-stationary economic growth by the three methods, and Figure 4 shows the RMSE comparison of the 100-time estimation results of the three methods. It can be seen from Figure 3 and Figure 4 that the three methods can track the real state, but the RMSE of the CRPF and MH-CRPF (Intelligent CRPF) is larger than that of the proposed method. Table 1 shows RMSE and MAE of 100-time estimation results of the mathematical model of one-dimensional non-stationary economic growth by the three methods. According to Table 1, compared with the other two comparison methods, the RMSE and MAE of the method in this paper are both minimal. According to these experimental results, the proposed method in this paper adopts a resampling strategy based on multi-population cooperation. It strengthens the interaction of high-weight particle information between different populations and reduces the dependence of the algorithm on the initial state value. This strategy can improve the diversity of the particles and the accuracy of the CRPF. Therefore, compared with the standard CRPF and MH-CRPF (Intelligent CRPF), the proposed method has higher estimation accuracy when the initial distribution of the particles is unknown.

### 4.2. One-Dimensional Nonlinear Univariate Time Series Model

The one-dimensional nonlinear univariate time series model is also widely used to verify the effectiveness of the particle filter due to its strong nonlinearity. The model is described as follows:

(16) $$x_{k}=1+\sin\left(0.04\pi t\right)+0.5x_{k-1}+w_{k}$$

(17) $$y_{k}=\left\{\begin{array}{cc} 0.2x_{k}^{2}+v_{k} & k\leq 30\\ 0.5x_{k}-2+v_{k} & k>30 \end{array}\right.$$

where $w_{k}∼N\left(0,\sigma_{w}^{2}\right)$ and $v_{k}∼N\left(0,\sigma_{v}^{2}\right)$ are process noise and observation noise, respectively.

In this simulation, $\sigma_{\omega }^{2}$, $\sigma_{v}^{2}$ and the initial values $x_{0}$ of $x_{k}$ as well as other parameter settings are consistent with those in Section 4.1.

**Figure 5:**
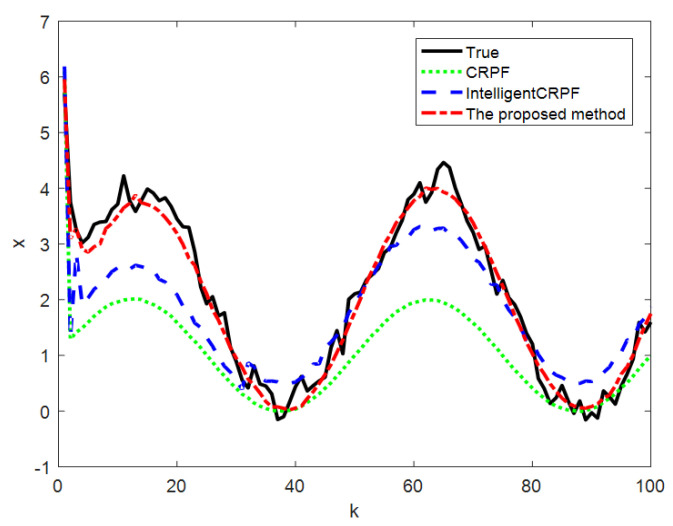
Comparison of three methods for state estimation of model Section 4.2.

**Figure 6:**
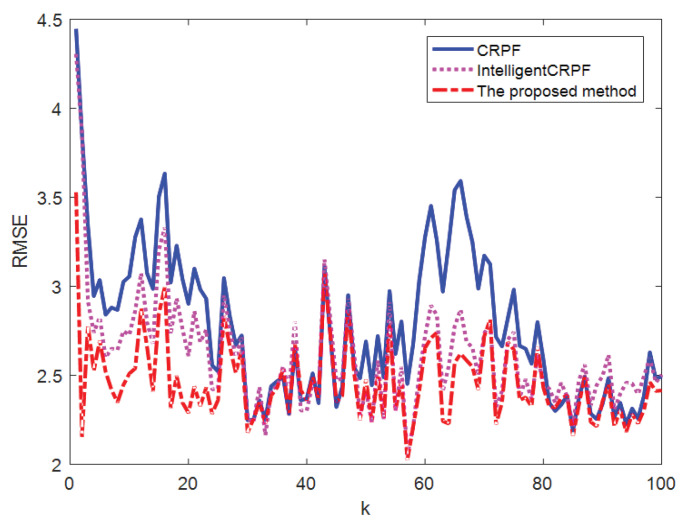
RMSE of state estimation results of three methods of model Section 4.2.

Figure 5 shows the comparison of the average values of the 100-time state estimation results of the one-dimensional nonlinear univariate time series model by the three methods. Figure 6 shows the RMSE comparison of the 100-time state estimation results of the three methods. It can be seen from Figure 5 and Figure 6 that the proposed method can track the real state well, but MH–CRPF (Intelligent CRPF) has a certain error in the estimation process, while the CRPF has a large error. Table 2 shows the RMSE and MAE of 100 independent repeated experiments estimated by the three methods for the one-dimensional nonlinear univariate time series model. As can be seen from Table 2, the RMSE and MAE of the proposed method are always the smallest, followed by MH-CRPF (Intelligent CRPF) and the largest CRPF. Therefore, when the initial state value is unknown, the state estimation accuracy of the proposed method for the one-dimensional nonlinear univariate time series model is higher than that of the other two methods, due to the multi-population intelligent cooperative resampling mechanism.

### 4.3. Lithium–Ion Battery Remaining Useful Life Prediction Model

Due to the high-dimensional, nonlinear and non-Gaussian characteristics of the remaining useful life prediction model of a lithium–ion battery, the nonlinear filtering algorithm is usually used to predict the remaining life of a lithium–ion battery. Therefore, this model can also be used to verify the effectiveness of the filtering algorithm. The model is described below.

(18) $$x_{k}=x_{k-1}+w_{k}$$

(19) $$Q_{k}=a_{k}\exp\left(b_{k}*k\right)+c_{k}\exp\left(d_{k}*k\right)+v_{k}$$

(20) $$x_{k-1}=\left[\begin{array}{c} a_{k-1}\\ b_{k-1}\\ c_{k-1}\\ d_{k-1} \end{array}\right]$$

where *k* is the number of cycles of a lithium–ion battery, $Q_{k}$ is the capacity of the lithium–ion battery at the k-th cycle, $w_{k}∼N\left(0,\sigma_{w}^{2}\right)$ and $v_{k}∼N\left(0,\sigma_{v}^{2}\right)$ are process noise and observation noise, respectively.

This simulation selects the data of the B0007 lithium–ion battery, which is widely used in the verification experiment of lithium battery remaining life prediction [26]. These data from NASA laboratories are common to international simulation experiments. In this simulation, the population number and particle number are the same as in Section 4.1, the rate of Gaussian mutation is $\sqrt{0.05}$, the particle number of the other two methods is 240 and the process variances are all U(0,1.5) random numbers.

**Figure 7:**
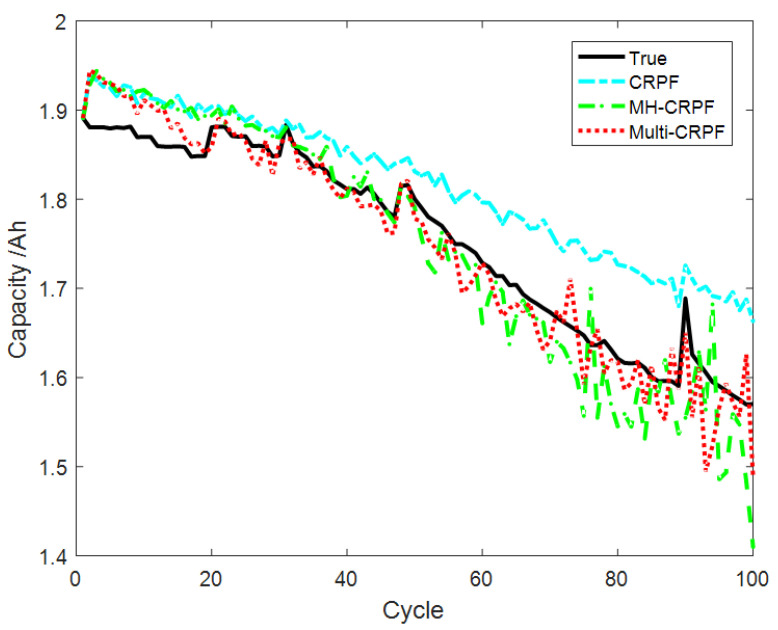
Prediction results of model Section 4.3 by three methods.

**Figure 8:**
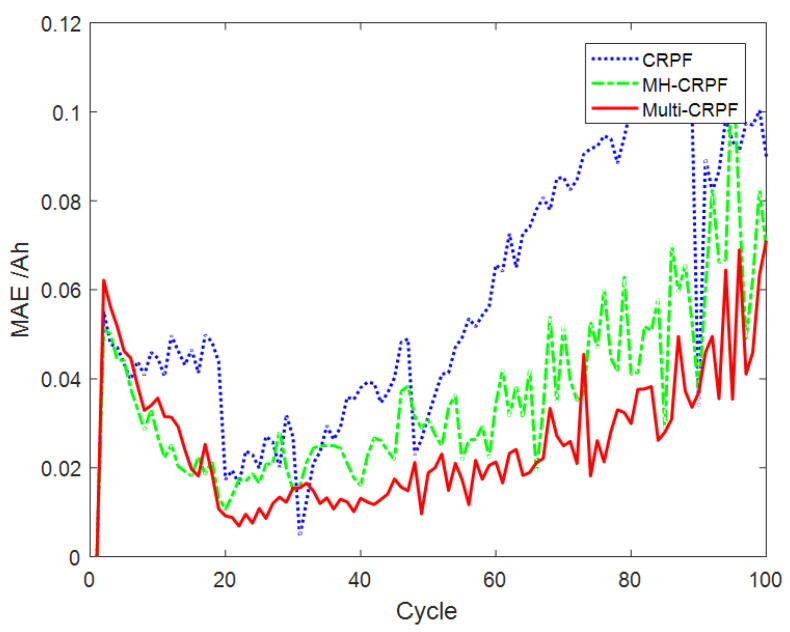
Comparison of state estimation MAE by three methods of model Section 4.3.

Figure 7 shows the comparison curve of prediction results of 100 independent repeated experiments on the remaining life of the B0007 lithium battery by the three methods. It can be seen from Figure 7 that the three methods can track the actual curve well. However, with the increase in the service cycles, the CRPF and MH-CRPF gradually deviate from the actual curve. The proposed method in this paper can always track the actual curve well. Figure 8 shows the comparison of the MAE of the remaining life prediction results of the B0007 lithium battery by the three methods. As can be seen from Figure 8, the deviation between the CRPF and MH-CRPF keeps increasing with the increase in time. The deviation of the proposed method (Multi-CRPF) is smaller than that of the other two methods. The proposed method shows better prediction accuracy. Table 3 shows the RMSE and MAE of 100 independent repeated experiments predicted by the three methods for the remaining life of the B0007 lithium battery. As can be seen from Table 3, the RMSE and MAE of the predicted results of the proposed method are both smaller than those of the other two methods. It can be concluded that, compared with the standard CRPF and MH-CRPF, the proposed method has better performance in predicting the remaining useful life of lithium batteries, especially when the initial value of the system state is unknown.

According to the above three simulation results, each population uses different initial state value to generate particles, and the multi-population cooperative intelligent resampling mechanism based on ring structure and Gaussian mutation strategy is proposed in this paper. It can effectively reduce the dependence of state estimation results on the initial state value. In addition, the high-weight particle information of each population is shared to other populations based on the ring structure. The high-weight and medium-weight particles of each population are retained, and the low-weight particles are resampled, which can effectively improve the diversity of the particles, so as to improve the estimation accuracy of the CRPF.

## 5. Conclusions

When the statistical characteristics of noise are unknown, the CRPF shows good filtering performance. An intelligent CRPF method based on multi-population cooperation is proposed to solve the problem of low estimation accuracy caused by the lack of particle diversity in the CRPF when the initial value of the particles is not set accurately.

The main contribution of this method is that the multi-population cooperative intelligent resampling method based on ring structure and the cooperative strategy based on Gaussian mutation are introduced into the CRPF. The particles are divided into several independent populations with different initial state values, and after the importance sampling is completed, the resampling process of low-weight particles is improved based on the ring structure. This method promotes the cooperation of particles among various populations and enhances the interaction ability of particle information. At the same time, Gaussian mutation is introduced to improve the particle diversity. The results of three simulation experiments show that, compared with the standard CRPF and MH-CRPF, the proposed method can effectively improve the estimation accuracy of the CRPF when the initial value of the state is not set accurately.

Although the proposed method has good estimation results, how to better set the particle initial value of each population and the mutation rate of Gaussian mutation, and how to enrich the cooperative strategy between multiple populations, so as to achieve more accurate estimation results, are the future research directions and research goals.

## Figures and Tables

**Table 1:** State estimation results of the three methods of model Section 4.1.

Performance Index	Multi-CRPF (Proposed)	MH-CRPF (IntelligentCRPF)	CRPF
RMSE	2.6220	3.2517	4.2156
MAE	1.9961	2.5345	3.5527

**Table 2:** State estimation results of the three methods of model Section 4.2.

Performance Index	Multi-CRPF (Proposed)	MH-CRPF (IntelligentCRPF)	CRPF
RMSE	2.4747	2.6284	2.8030
MAE	1.8861	2.0164	2.1852

**Table 3:** Prediction results of remaining useful life of lithium battery by three methods.

Performance Index	Multi-CRPF (Proposed)	MH-CRPF (IntelligentCRPF)	CRPF
RMSE	0.0363	0.0503	0.0646
MAE	0.0258	0.0360	0.0574

## Data Availability

Not applicable.
